# Plant accession and insect infestation, rather than silicon supplementation, shape defence strategies of *Arabidopsis halleri* towards a leaf beetle

**DOI:** 10.1111/plb.70160

**Published:** 2025-12-22

**Authors:** R. Putra, M. Paulic, C. Müller

**Affiliations:** ^1^ Department of Chemical Ecology Bielefeld University Bielefeld Germany; ^2^ Department of Ecology, Institute of Entomology Biology Centre of the Czech Academy of Sciences České Budějovice Czech Republic

**Keywords:** Glucosinolate, herbivory, induced defence, intraspecific variation, trichome

## Abstract

- Little is known about the functional role of silicon (Si) in metal‐hyperaccumulating plant species, such as *Arabidopsis halleri*. We investigated the responses of *A. halleri* from two accessions, Bestwig (Best) and Langelsheim (Lan), to Si supplementation and insect infestation in two controlled full‐factorial experiments.
- Plants were grown in soil either unsupplemented (−Si) or supplemented (+Si) with Si. Some of these plants were kept either uninfested or infested by larvae of the leaf beetle *Phaedon cochleariae*. Shoot chemical and mechanical traits and plant resistance against the larvae were quantified. Detached leaves from the remaining plants were used to examine whether trichome density and leaf area consumed by larvae were influenced by the accession and/or Si.
- We found that Si supplementation, but not insect infestation or their interaction, led to twice as high concentrations of shoot Si in +Si in comparison to −Si plants. Insect relative growth rate was not impacted by Si, but by accession, namely lower when larvae fed on Lan than on Best plants. Likewise, leaf area consumed by larvae was consistently lower in the former accession. The density of trichomes was twice as high in plants of the Lan than the Best accession. Uninfested +Si plants contained the highest C/N in both accessions. The composition of glucosinolates differed between accessions, with some glucosinolates being induced by Si, insect infestation or both in the Best plants only.
- Our findings highlight distinct (induced) defence strategies within *A. halleri* plants, which may indicate different local adaptations of the source populations.

## Graphical abstract

Chemical and mechanical defences of a metal‐hyperaccumulating plant species *Arabidopsis halleri* were more influenced by plant accession (genetic background) and insect herbivory by a leaf beetle *Phaedon cochleariae* than by supplementation of the metalloid silicon.

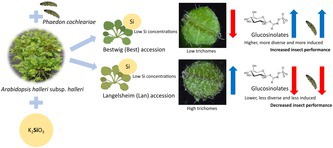

## INTRODUCTION

Besides acquiring carbon (C), oxygen (O) and hydrogen (H) for photosynthesis, land plants must take up other elements or nutrients particularly from the soil, such as nitrogen (N), phosphorus (P), potassium (K), including some metals and metalloids (Brown *et al*. 2022). Uptake and accumulation of certain metals and metalloids may be used to support plant metabolism and physiology. A long‐neglected class of plant nutrients, namely ‘beneficial elements’ (Brown *et al*. 2022), which include the metalloid silicon (Si), is nowadays increasingly studied, because Si plays numerous roles in abiotic and biotic stress amelioration (Coskun *et al*. 2019) most notably in Poaceae. However, less is known about the interplay between Si and herbivory as biotic stress on plant responses in taxa other than Poaceae.

Once taken up, Si is often accumulated and deposited in the cell walls, within and between cells or apoplasts of roots and shoots (Epstein 1999). Many plants contain Si in the shoots, ranging from 0.1% to 10% on a dry mass basis (Epstein 1999). Moreover, Si supplementation is known to alter some plant phenotypic traits. For example, leaf surface structures, such as silica and prickle cells, were significantly larger in Si‐supplemented than unsupplemented plants of *Brachypodium distachyon* (Hall *et al*. 2020). The thickness of the leaf epidermis and the cuticle as well as trichome densities significantly increased with Si relative to plants without Si supplementation in *Cucumis sativus*, *C. melo* and *Capsicum annuum* (Ferrón‐Carrillo & Urrestarazu 2021). With regard to relationships between element contents predominantly in high Si‐accumulating species, the trade‐off hypothesis initially postulates a negative correlation between leaf Si and leaf C from the point of view of lower energetic costs to incorporate Si than lignin or cellulose as a structural component in plants (Raven 1983). Then, other previous studies also found similar negative relationships between leaf Si and organic defence compounds (*e.g*., phenolics) and lignin‐derived phenols (Johnson & Hartley 2018; Klotzbücher *et al*. 2018; Hodson & Guppy 2022). Relationships between leaf Si and N are less clear‐cut across plant species, but at least in some Fabaceae species, their contents are positively correlated (Putra *et al*. 2020, 2022). Plant Si‐mediated changes in some functional traits are therefore expected to impact plant interactions with the (biotic) environment, including insect herbivores.

Silicification in some plants has been shown to exert direct consequences for insect herbivores. Negative impacts on insect mandibles, feeding efficiencies, growth rates and immune responses are some examples of how silicification can confer a potent defence against leaf‐chewing insects mechanistically (Massey & Hartley 2009; Waterman *et al*. 2021; Cibils‐Stewart *et al*. 2023). While direct mechanisms by which silicification can protect some plants against herbivory are well documented, little is known about Si effects on organic plant defences that may impact herbivores. Albeit with speculative findings, a previous study summarised that Si supplementation of *B. distachyon* plants led to decreases in the concentrations of phytohormone jasmonic acid (JA), following herbivory stress, probably because they already invested in mechanical defences (*e.g*., macro‐hairs) and physical defences (*e.g*., silicified cells) (see Fig. 2 in Hall *et al*. 2019). Whether these findings remain consistent in other plant species, further investigations are required. Likewise, herbivory affects the quantity and quality of plant traits, not only causing tissue losses but also inducing changes in various plant traits (Salgado‐Luarte & Gianoli 2012; Underwood 2012). Previous studies revealed that plant Si as an elemental defence can be further induced by herbivory particularly in high Si‐accumulating species (Johnson *et al*. 2020; Waterman *et al*. 2021). In the metal‐hyperaccumulating Brassicaceae species *Arabidopsis halleri* (meadow rock‐cress), metals, such as cadmium (Cd) and zinc (Zn), increased in concentrations in leaf phloem exudates upon infestation by the aphid *Myzus persicae* (Sulzer, 1776) (Hemiptera: Aphididae) (Stolpe *et al*. 2017). To our knowledge, it is still unknown how Si supplementation may impact plant traits in combination with insect infestation and influence insect feeding preferences in this species.

*Arabidopsis halleri* is a close relative of the model plant *A. thaliana* (Clauss & Koch 2006) and can grow on a wide range of soil conditions (Rellstab *et al*. 2017; Stein *et al*. 2017; Buckley *et al*. 2019) covering areas of Europe and East Asia (Honjo & Kudoh 2019). Thus, *A. halleri* plants are also very likely exposed to available Si in soils. Si was found to co‐precipitate with Zn as Zn‐silicate inside the vacuole in leaf cells of *A. halleri* from one accession (Langelsheim, Germany), suggesting a potential role of Si in Zn detoxification (Neumann & De Figueiredo 2002). Furthermore, Si supplementation has been shown to affect mechanical traits of *A. halleri*, that is, trichome density, in a plant accession‐specific manner (Putra *et al*. 2024). As a Brassicaceae, *A. halleri* also contains glucosinolates as characteristic specialised metabolites, which significantly vary in composition and concentration among plant populations and accessions of *A. halleri* (Kazemi‐Dinan *et al*. 2015; Putra *et al*. 2024). These mechanical and chemical defences should have consequences on plant–insect interactions. In the field, *A. halleri* may be attacked by different insect species, such as the oligophagous leaf beetle *Phaedon brassicae* (Baly, 1874) (Coleoptera: Chrysomelidae), known to infest *A. halleri* subsp. *gemmifera*, native to the Russian Far East and East Asia, including Japan. In Germany, *A. halleri* subsp. *halleri* plants grow in open forest and creek areas, where a close relative of *P. brassicae*, *Phaedon cochleariae* (Fabricius, 1792) (Coleoptera: Chrysomelidae), can co‐occur.

Here, we investigated the potential interplay between Si supplementation and insect infestation on various traits in *A. halleri* subsp. *halleri* and studied the impacts of plant accession and Si supplementation on insect feeding preferences, using *P. cochleariae* as the study organism. We selected plants from two accessions originating from two areas in Germany, namely Bestwig (Best) and Langelsheim (Lan), which present two distinct glucosinolate leaf chemotypes (Kazemi‐Dinan *et al*. 2015), and conducted two full‐factorial experiments. We mainly aimed to examine the effects of Si supplementation and insect infestation on shoot chemical traits and insect relative growth rate (RGR) as a proxy for plant resistance. Moreover, insect feeding preferences in a two‐choice detached leaf bioassay and trichome density as a proxy for leaf mechanical defence were investigated. We hypothesized that Si supplementation and insect infestation independently influence shoot chemical traits, with herbivory exerting more pronounced effects than Si because of direct leaf damage by the herbivores. Larval RGR was expected to be higher when the larvae fed on plants without Si than with Si supplementation in both accessions, likely due to lower Si concentrations in plants without Si supplementation. Likewise, the larvae may prefer choosing and feeding on leaves with lower Si concentrations. However, trichome density may be influenced by Si and plant accession, and hence, leaf mechanical defence could presumably change the directionality and magnitude of larval performance and preference.

## MATERIALS AND METHODS

### Plant and insect rearing

Plants of *A. halleri* were collected in two areas in Germany, namely Bestwig (‘**Best**’, hereafter) and Langelsheim (‘**Lan**’, hereafter), and then grown in controlled greenhouse conditions for a few years (Stein *et al*. 2017). For this current experiment, plants were clonally propagated from shoot cuttings (n = 62 per accession) and grown in pots with a mixture of soil (‘type P’, low nutrient) and sand (2:1, Hawita, Germany) in a greenhouse (70% r.h., 20°C and 8:16 h light:dark photoperiod with an automatic shading system) as described previously in Putra *et al*. (2024). This substrate was steam‐sterilised and contained low concentrations of bioavailable Si (0.077 ± 0.005 mg/g, mean ± SE; n = 9). Pots were irrigated with 5 ml of demineralised water once a week for the first 37 days. Afterwards, the 4‐week‐old plants including the substrate were transplanted into larger pots (70 × 70 × 80 mm) with the same substrate and grown in a controlled growth chamber (Percival, CLF Plant Climatics, Wertingen, Germany) at 60% r.h., 20°C and 8:16 h light:dark photoperiod. The plants were irrigated with demineralised water twice a week before Si supplementation.

Subsequently, half of the 5‐week‐old plants were supplemented twice a week with 5–10 ml KCl solution (1.7 mM; AppliChem, Darmstadt, Germany) and assigned as **−Si**, while the other half of the plants were supplemented with 5–10 ml of K_2_SiO_3_ solution (2 mM; Carl Roth, Karlsruhe, Germany) and assigned as **+Si** (amounts were increased over time). The pH of the K_2_SiO_3_ solution was adjusted with 32% [*v/v*] of diluted HCl (Fisher Scientific, Loughborough, UK) to be similar to the pH of the KCl solution (pH = 6.74). The solutions were prepared following an established method as described in Putra *et al*. (2021). After 7 weeks under these treatments, the 13‐week‐old plants were assigned to two separate full‐factorial experiments to examine the effects of: (1) Si supplementation and insect infestation on shoot chemical traits (Experiment 1) and (2) accession and Si supplementation on leaf phenotypic traits and insect feeding preferences (Experiment 2).

Individuals of *P. cochleariae* were reared for several generations in a climate cabinet (70% r.h., 20°C, 16:8 h light:dark photoperiod) and provided with leaves of Chinese cabbage (*Brassica rapa* subsp. *pekinensis*) prior to bioassays. For the experiments, second‐instar larvae were used, from which growth rates can be well determined, as they continuously gain body mass, which is crucial for their development (Tanton 1977; Reifenrath & Müller 2008).

### Experiment 1

**Fig. 1:**
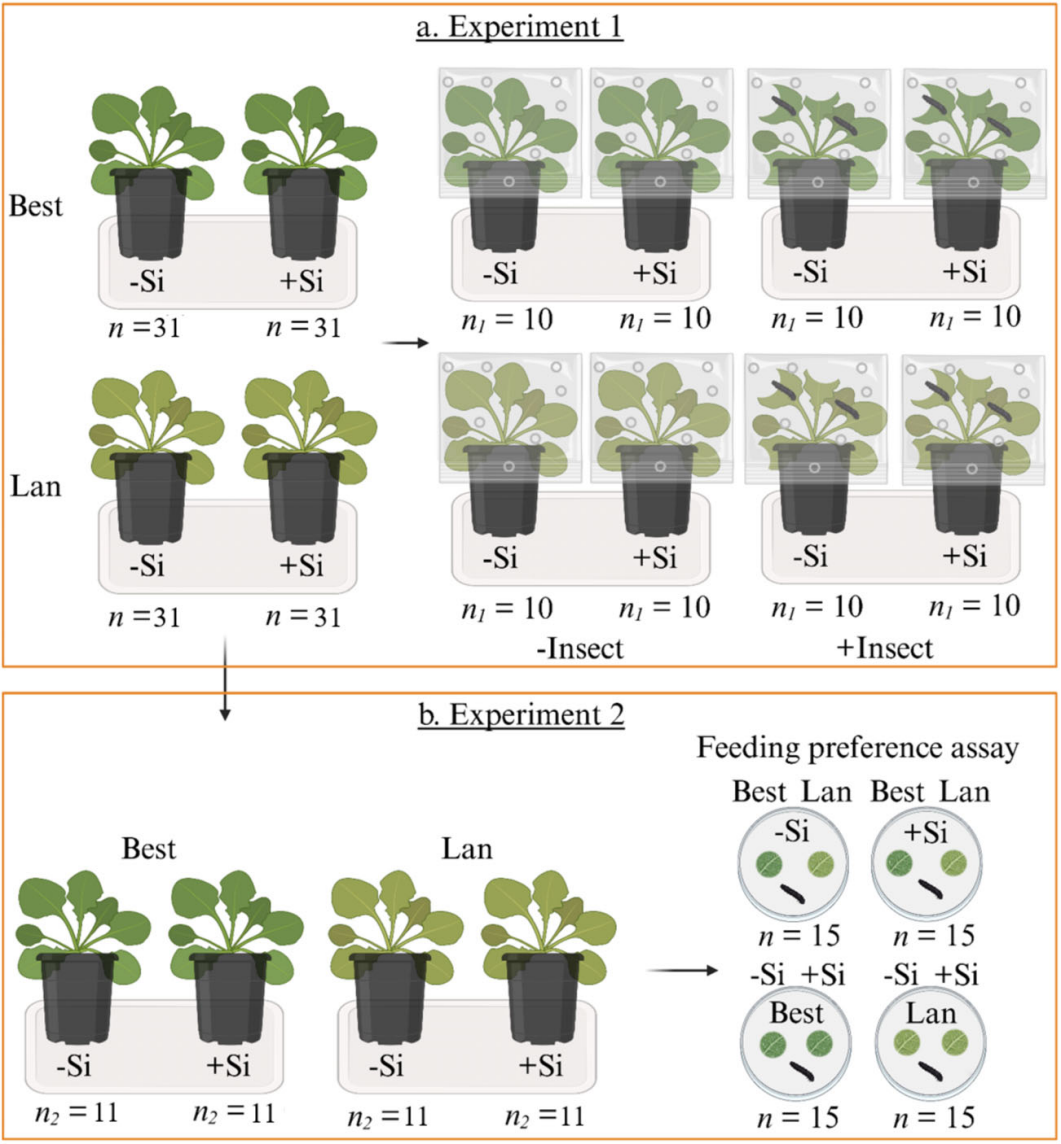
Design for: (a) Experiment 1 to examine the effects of silicon (Si) supplementation and insect infestation on shoot chemical traits of *Arabidopsis halleri* accessions [Bestwig (Best) and Langelsheim (Lan)] growing in soil without (−Si) and with Si (+Si) supplementation and kept uninfested (−Insect) or infested (+Insect) by two larvae of *Phaedon cochleariae* and (b) Experiment 2 to investigate the effects of accession and Si supplementation on leaf mechanical traits and insect feeding preferences of *P. cochleariae* larvae; *n* indicates the number of replicates for each experiment. The figure was created with BioRender.com.

To investigate the effects of Si supplementation and insect infestation on shoot chemical traits, plants of the −Si and +Si from each of the two accessions, Best and Lan, were kept either uninfested (**−Insect**) or infested by two larvae of *P. cochleariae* (**+Insect**) for a week, resulting in eight treatment combinations (Best −Si −Insect; Best −Si +Insect; Best +Si −Insect; Best +Si +Insect; Lan −Si −Insect; Lan −Si +Insect; Lan +Si −Insect; and Lan +Si +Insect) with *n* = 10 initial replicates per combination (Fig. 1a). All pots were covered with perforated transparent plastic bags to prevent larvae from escaping. After a week of infestation, all larvae were gently removed and shoots were cut above the soil surface, cleaned from insect frass, frozen in liquid N, stored at −80°C and lyophilised. Dried shoot materials were homogenised in a ball mill for further chemical analyses. Before and after the infestation, the fresh mass of the groups of two larvae was recorded to determine the RGR of the insects as a measure of plant resistance. Insect RGR was calculated based on the difference between the larval fresh mass divided by the number of infestation days (*i.e*., 7) and divided by the number of larvae (*i.e*., 2).

### Elemental analysis

Shoot C and N contents were quantified from subsamples of homogenised shoot material (approximately 2.5 mg dw per sample) by a combustion method with a micro‐elemental analyser (CN‐vario MICRO cube; Elementar Analysensysteme GmbH, Hanau, Germany). Five samples of acetanilide (Merck KGaA, Darmstadt, Germany) as calibration standards were prepared in the same way for quantification. Shoot Si concentrations were determined (from about 20 mg dw per sample) using the molybdenum‐blue colorimetric method as described in Nakamura *et al*. (2020) and spectrophotometrically quantified at 650 nm (Thermo Scientific Multiskan FC, Schwerte, Germany) in a 96‐well microplate against a blank and a standard curve of Si (ICP‐MS grade; Merck KGaA, Darmstadt, Germany).

### Glucosinolate analysis

Subsamples of the shoot material (approximately 15 mg dw per sample) were extracted threefold in 80% [*v/v*] methanol (500 μl each), using *p*‐hydroxybenzyl glucosinolate (Phytoplan Diehm & Neuberger, Heidelberg, Germany) as an internal standard. Centrifugation of the samples was performed at 2700 × g for 10 min at room temperature. After glucosinolates were converted to desulfoglucosinolates following the established method as in Barber & Müller (2021), samples were analysed using high‐performance liquid chromatography coupled with a diode array detector (Dionex Ultimate 3000; Thermo Fisher Scientific, Waltham, MA, USA). Glucosinolates were quantified relative to the amount of the internal standard with integrated peak areas at 229 nm by comparing their retention times and spectra against an in‐house databank, taking response factors into account, and in relation to the sample dry mass.

### Experiment 2

To study the effects of Si supplementation on leaf mechanical traits and insect feeding preferences, −Si and +Si plants from each of the two accessions, Best and Lan, were used, resulting in four treatment combinations in total (Best −Si; Best +Si; Lan −Si; and Lan +Si) with n = 11 initial replicates per combination (Fig. 1b). To determine different leaf mechanical traits and insect feeding preferences, middle‐aged leaves were detached from the 13‐week‐old plants.

One leaf disc (4 mm diameter, 12.56 mm^2^ area) per plant was taken to count the number of trichomes on the adaxial and abaxial sides under a stereomicroscope (Olympus SZX16, Tokyo, Japan). The number of trichomes from both sides was summed up per sample.

Further leaf discs were offered in insect feeding preference assays. Therefore, discs were weighed individually and placed in pairs, testing leaf discs of either both Si treatments within accession or the two accessions within Si treatment against each other. Two discs were placed onto a moistened filter paper inside a Petri dish (50 mm diameter) at a distance of 20 mm with n = 15 replicates per combination (Fig. 1b). One larva of *P. cochleariae* was placed in the centre of each dish and removed after 24 h. Afterwards, the remaining leaf discs were placed on millimetre paper, scanned and analysed using ImageJ/Fiji (Schindelin *et al.* 2012) to determine the consumed leaf area.

### Statistical analyses

All statistical analyses were performed in R version 4.3.3 (R Core Team 2024). In Experiment 1, a three‐way analysis of variance (ANOVA) in the ‘lm’ function with type II was used for continuous data, whose residual models fulfilled the assumptions for normality by quantile–quantile plots (‘qqPlot’) and Shapiro–Wilk's test. Homoscedasticity was determined by Levene's test. If residual models did not fulfil, such assumptions, data transformation by sqrt or log_*e*_ was applied and transformed data were re‐analysed in the ‘lm’ function. If residual models of transformed data still did not fulfil these assumptions, then the two‐way generalised linear model in the ‘glm’ function with the ‘gaussian’ family, followed by the ‘Anova’ function with type II, was used for non‐parametric data. The functions ‘lm’, ‘qqPlot’ and ‘glm’ were obtained from the ‘car’ package (Fox & Weisberg 2019). In cases of statistical differences for interaction between two or among three factors, Tukey's HSD tests were computed to examine which group treatments differed significantly using ‘pairs’ followed by ‘cld’ functions from the ‘multcomp’ package (Hothorn *et al*. 2008) according to marginal means of the models (‘emmeans’).

In Experiment 2, plant accession (Best or Lan) and Si supplementation (−Si or +Si) and their interaction were set as main factors, whereas a leaf mechanical trait, that is trichome density, was set as a response variable. Similar to above, the ‘lm’ or ‘glm’ function was applied to analyse all response variables depending on whether residual models fulfilled the assumptions of normality and homoscedasticity. Density of total trichomes was analysed using the ‘glm’ function with the ‘poisson’ family because they were discrete (count) data. Wilcoxon rank‐sum tests from the ‘stats’ package were performed to analyse consumed leaf area as a measure for insect feeding preferences between plant accession and Si supplementation.

In Experiment 1, to visualise the composition of glucosinolates, principal component analysis (PCA) was performed across all samples using ‘prcomp’ [the ‘ggfortify’ package in Tang *et al*. 2016; the ‘devtools’ package in Wickham *et al*. 2022]. To examine whether each of the factors plant accession (Best or Lan), Si supplementation (−Si or +Si) and insect infestation (−Insect or +Insect), as well as their three‐way interaction, contributed significantly (*P* < 0.05) to the overall composition of glucosinolates, permutational multivariate analyses of variance (PERMANOVA) were performed using ‘adonis’ from the ‘vegan’ package with the Bray–Curtis dissimilarity (Oksanen *et al*. 2024). PERMANOVA was selected here because the residual model of glucosinolate composition was non‐parametric. PERMANOVA indicated that accession contributed significantly (*F* = 140.42, *P* = 0.001) to responses in the composition of glucosinolates (Fig. 7a). Data visualisation in both experiments was generated using ‘ggplot’ from the ‘ggplot2’ package (Wickham 2009).

## RESULTS

### Plant resistance of *A. halleri* against *P. cochleariae* was more impacted by the accession than Si supplementation

**Fig. 2:**
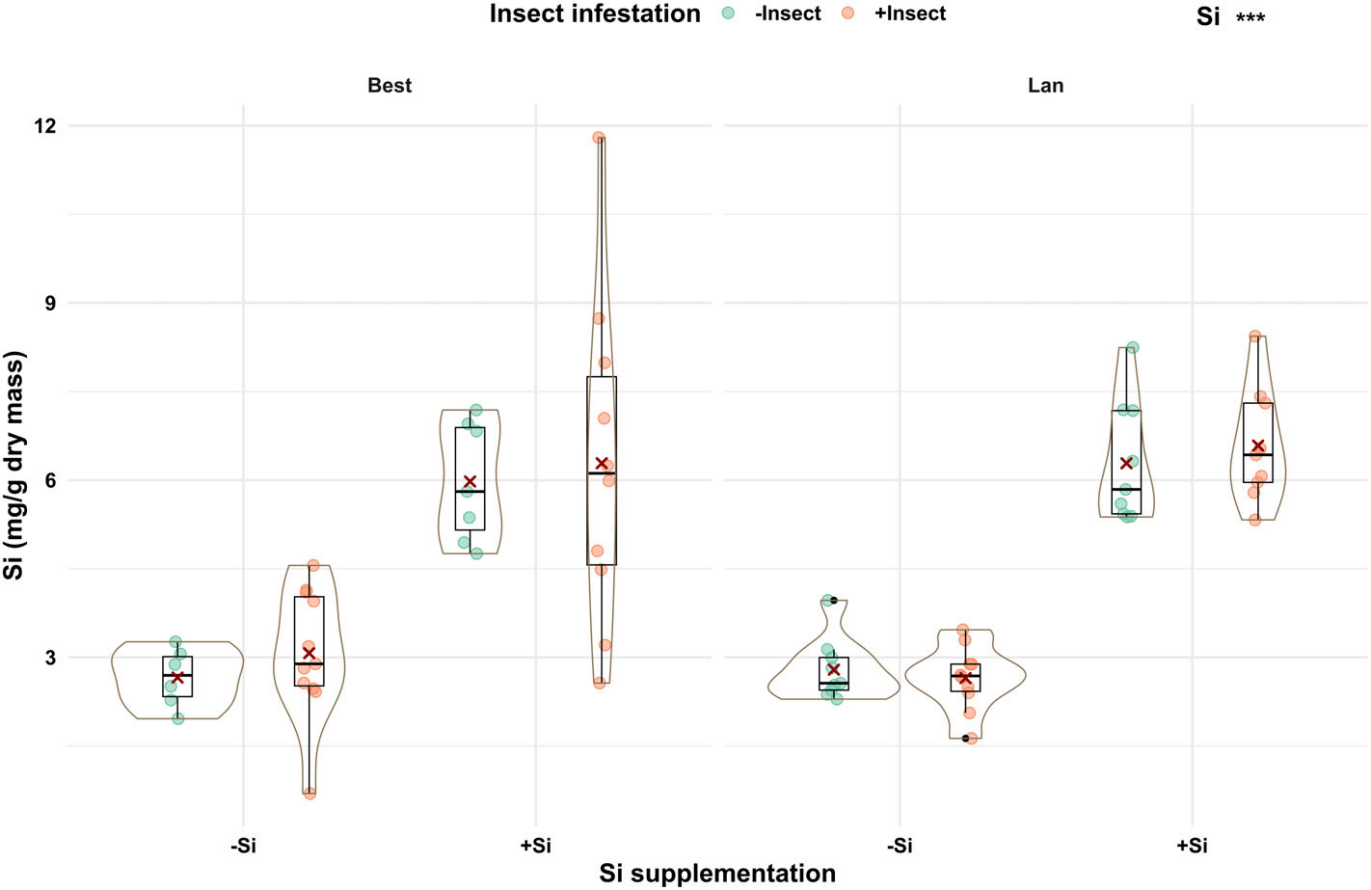
Shoot silicon (Si) concentrations of *Arabidopsis halleri* plant accessions [Bestwig (Best) and Langelsheim (Lan)] grown on soil without (−Si) or with Si (+Si) supplementation and kept uninfested (−Insect) or infested (+Insect) by *Phaedon cochleariae* larvae. Solid circles represent data points; solid dots, outliers; solid lines, medians; crosses, the means; boxes, the interquartile ranges; and whiskers, the 5% and 95% percentiles surrounded by violin plots indicating the width of data distribution. Statistical significance is shown as ***P < 0.001.

**Fig. 3:**
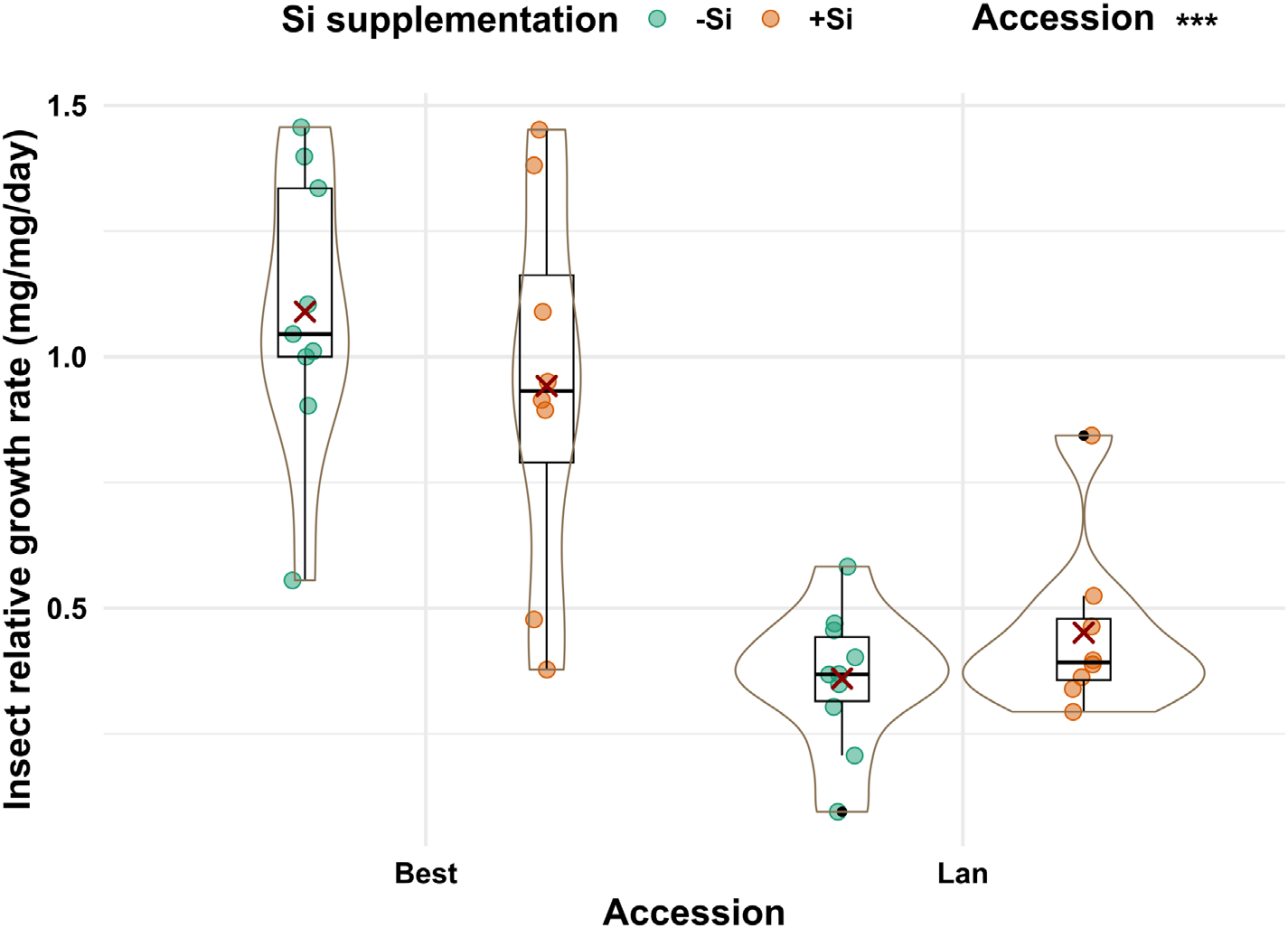
Insect relative growth rate of *Phaedon cochleariae* larvae as a proxy for plant resistance when fed on two accessions [Bestwig (Best) and Langelsheim (Lan)] of *Arabidopsis halleri* plants grown on soil without (−Si) or with Si (+Si). Solid circles represent data points; solid dots, outliers; solid lines, medians; crosses, the means; boxes, the interquartile ranges; and whiskers, the 5% and 95% percentiles surrounded by violin plots indicating the width of data distribution. Statistical significance is shown as ***P < 0.001. Si, silicon.

In both accessions, shoot concentrations of Si were significantly lower in −Si than +Si plants, regardless of insect infestation, by twofold (Fig. 2; Table 1). The RGR of the larvae, taken as a measure of plant resistance, was significantly impacted by accession (df = 1, *F* = 51.58, *P* < 0.001), but not by Si supplementation (df = 1, *F* = 0.01, *P* = 0.92) or their interaction (df = 1, *F* = 1.9, *P* = 0.18). The RGR was 2.5 times higher for larvae feeding on plants of the Best than Lan accession (Fig. 3), indicating that plants of the former accession were more susceptible to the larvae than those of the latter accession.

**Table 1:** Effects of main factors and their interactions on shoot chemical traits in *Arabidopsis halleri* plants based on a three‐way linear (*F*) or generalised linear (χ^2^) model.

Response variables	Factors																				
	Accession			Si			Insect			Accession × Si			Accession × Insect			Si × Insect			Accession × Si × Insect		
	**df**	**χ** ^**2**^	***P***	**df**	**χ** ^**2**^	***P***	**df**	**χ** ^**2**^	***P***	**df**	**χ** ^**2**^	***P***	**df**	**χ** ^**2**^	***P***	**df**	**χ** ^**2**^	***P***	**df**	**χ** ^**2**^	***P***
Si concentrations	1	0.037	0.847	1	20.585	<0.001***	1	0.383	0.536	1	0.033	0.856	1	0.381	0.537	1	0.013	0.91	1	0.186	0.666
C	1	2.615	0.106	1	1.013	0.314	1	4.209	0.04*	1	0.563	0.453	1	2.672	0.102	1	2.718	*0.099*	1	0.252	0.615
	**df**	***F***	***P***	**df**	***F***	***P***	**df**	***F***	***P***	**df**	***F***	***P***	**df**	***F***	***P***	**df**	***F***	***P***	**df**	***F***	***P***
N	1	11.95	<0.001***	1	0.021	0.884	1	0.429	0.515	1	0.626	0.432	1	0.188	0.666	1	13.997	<0.001***	1	0.647	0.424
C/N	1	9.103	0.004**	1	0.017	0.897	1	0.801	0.374	1	0.409	0.525	1	0.039	0.844	1	13.864	<0.001***	1	0.035	0.851
	**df**	***F***	***P***	**df**	***F***	***P***	**df**	***F***	***P***	**df**	***F***	***P***	**df**	***F***	***P***	**df**	***F***	***P***	**df**	***F***	***P***
5MSOP	1	60.756	<0.001***	1	0.518	0.474	1	0.183	0.67	1	0.176	0.676	1	2.673	0.107	1	0.624	0.433	1	4.558	0.037*
6MSOH	1	664.128	<0.001***	1	0.074	0.787	1	2.554	0.115	1	0.043	0.836	1	11.854	0.001**	1	3.111	*0.083*	1	4.563	0.037*
7MSOH	1	0.005	0.943	1	0.326	0.57	1	3.858	*0.05*	1	0.422	0.518	1	12.986	<0.001***	1	1.761	0.19	1	2.903	*0.094*
	**df**	**χ** ^**2**^	***P***	**df**	**χ** ^**2**^	***P***	**df**	**χ** ^**2**^	***P***	**df**	**χ** ^**2**^	***P***	**df**	**χ** ^**2**^	***P***	**df**	**χ** ^**2**^	***P***	**df**	**χ** ^**2**^	***P***
8MSOO	1	165.7	<0.001***	1	1.817	0.178	1	6.946	0.008**	1	1.358	0.244	1	9.629	0.002**	1	1.538	0.215	1	1.969	0.16
	**df**	***F***	***P***	**df**	***F***	***P***	**df**	***F***	***P***	**df**	***F***	***P***	**df**	***F***	***P***	**df**	***F***	***P***	**df**	***F***	***P***
6MTH	1	21.778	<0.001***	1	0.097	0.756	1	4.593	0.036*	1	0.3	0.586	1	4.02	0.049*	1	0.206	0.651	1	0.882	0.352
	**df**	**χ** ^**2**^	***P***	**df**	**χ** ^**2**^	***P***	**df**	**χ** ^**2**^	***P***	**df**	**χ** ^**2**^	***P***	**df**	**χ** ^**2**^	***P***	**df**	**χ** ^**2**^	***P***	**df**	**χ** ^**2**^	***P***
7MTH	1	69.114	<0.001***	1	0.069	0.792	1	6.572	0.01*	1	0.133	0.715	1	8.011	0.005**	1	0.326	0.568	1	0.423	0.515
8MTO	1	67.044	<0.001***	1	0.045	0.832	1	4.838	0.028*	1	0.029	0.864	1	5.885	0.015*	1	0.119	0.73	1	0.141	0.707
	**df**	***F***	***P***	**df**	***F***	***P***	**df**	***F***	***P***	**df**	***F***	***P***	**df**	***F***	***P***	**df**	***F***	***P***	**df**	***F***	***P***
Benzyl	1	15.641	<0.001***	1	0.061	0.805	1	2.581	0.114	1	0.508	0.479	1	2.66	0.108	1	8.195	0.006**	1	0.204	0.653
	**df**	**χ** ^**2**^	***P***	**df**	**χ** ^**2**^	***P***	**df**	**χ** ^**2**^	***P***	**df**	**χ** ^**2**^	***P***	**df**	**χ** ^**2**^	***P***	**df**	**χ** ^**2**^	***P***	**df**	**χ** ^**2**^	***P***
I3M	1	79.97	<0.001***	1	7.815	0.005**	1	2.364	0.124	1	8.166	0.004**	1	2.714	*0.099*	1	0.046	0.829	1	0.055	0.814
4MOI3M	1	82.894	<0.001***	1	2.269	0.132	1	0.129	0.72	1	1.791	0.181	1	0.026	0.873	1	0.881	0.348	1	1.403	0.236
4OHI3M	1	80.362	<0.001***	1	3.431	*0.064*	1	3.021	*0.082*	1	2.734	*0.098*	1	2.803	*0.094*	1	1.019	0.313	1	1.621	0.203
Total glucosinolates	1	5.006	0.025*	1	0.293	0.588	1	2.774	*0.096*	1	0.325	0.568	1	9.89	0.002**	1	0.515	0.473	1	1.116	0.291

*P*‐values in bold and in italic denote statistical significance and marginal significance, respectively. Statistically significant outcomes are shown as follows: ***P < 0.001, **P < 0.01, *P < 0.05, •*P* < 0.1 (marginally significant).

**Fig. 4:**
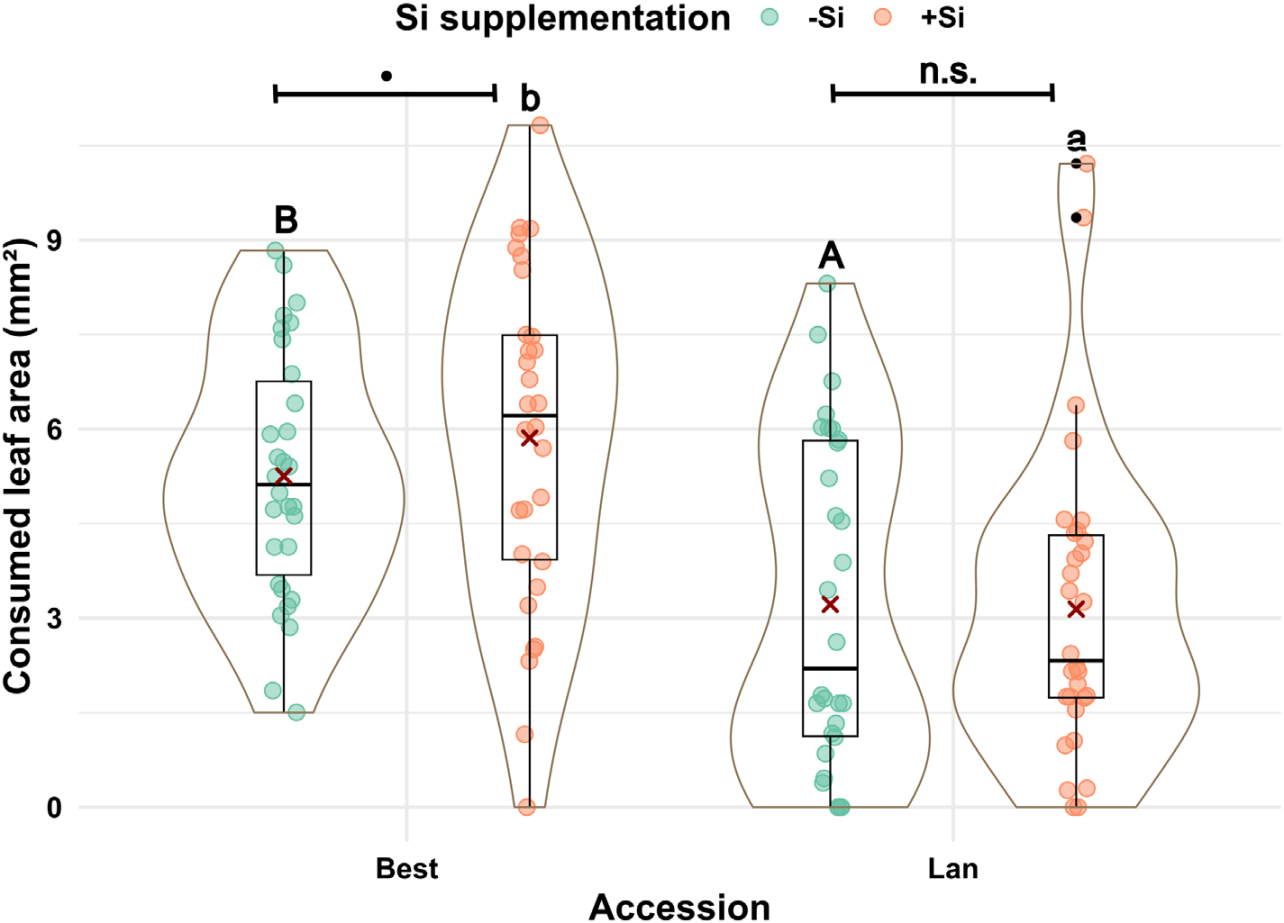
Mean leaf area (mm^2^) consumed by larvae of *Phaedon cochleariae* in two‐choice feeding preference assays using detached leaves of two accessions [Bestwig (Best) and Langelsheim (Lan)] of *Arabidopsis halleri* plants grown on soil without (−Si) or with Si (+Si) supplementation. Solid circles represent data points; solid dots, outliers; solid lines, medians; crosses, the means; boxes, the interquartile ranges; and whiskers, the 5% and 95% percentiles surrounded by violin plots indicating the width of data distribution. Different uppercase letters above plots indicate significant differences between *A. halleri* accessions without Si supplementation (−Si plants, green dots, *P* < 0.01). Different lowercase letters above plots indicate significant differences between *A. halleri* accessions with Si supplementation (+Si plants, orange dots, *P* < 0.01). Dot and n.s. above the plots denote marginal significance (*P* < 0.1) and non‐significant difference (*P* > 0.1), respectively, according to the Wilcoxon rank‐sum test. Si, silicon.

Consumed leaf area as a measure for insect feeding preferences by *P. cochleariae* larvae was more clearly impacted by accession than by Si treatment (Fig. 4). Regardless of Si supplementation, larvae consumed almost twofold more from leaves of the Best than from the Lan accession (Best −Si *versus* Lan −Si; *W* = 178, *P* = 0.007 and Best +Si *versus* Lan +Si; *W* = 196, *P* < 0.001). Within the Best accession, larvae consumed marginally more leaf material from +Si plants than from −Si plants (Best −Si *versus* Best +Si; *W* = 65, *P* = 0.05), while there was no statistical difference in consumed leaf area within the Lan accession (Lan −Si *versus* Lan +Si; *W* = 86, *P* = 0.28).

### Density of total leaf trichomes of *A. halleri* plants was impacted by accession × Si supplementation

**Fig. 5:**
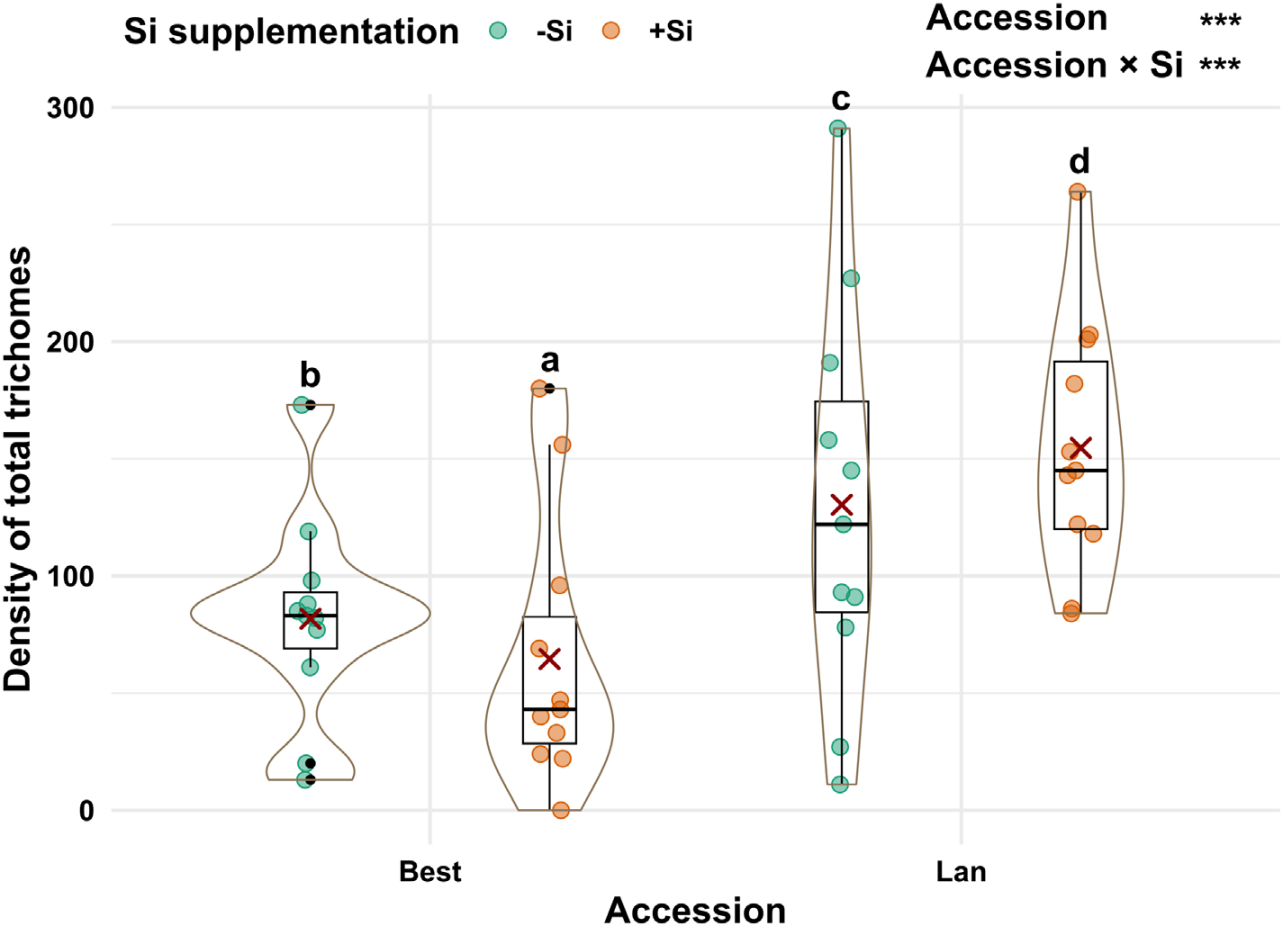
Density of total trichomes (adaxial and abaxial sides) per leaf disc area (12.56 mm^2^) in two accessions [Bestwig (Best) and Langelsheim (Lan)] of *Arabidopsis halleri* plants grown on soil without (−Si) or with Si (+Si) supplementation. Solid circles represent data points; solid dots, outliers; solid lines, medians; crosses, the means; boxes, the interquartile ranges; and whiskers, the 5% and 95% percentiles surrounded by violin plots indicating the width of data distribution. Statistical significance is shown as ***P < 0.001. Different letters above the plots indicate significant differences based on Tukey's HSD post hoc test. Si, silicon.

The density of total foliar trichomes was significantly impacted by accession (df = 1, χ^2^ = 499.7, *P* < 0.001) and accession × Si (df = 1, χ^2^ = 43.74, *P* < 0.001), but not by Si alone (df = 1, χ^2^ = 1.28, *P* = 0.26). Trichome density was on average twofold higher on leaves of plants of the Lan accession than on those of the Best accession (Fig. 5). Si supplementation resulted in decreases in total trichome densities in plants of the Best accession by on average 27%, but increases in plants of the Lan accession by 19%.

### Shoot C/N was influenced by Si supplementation × insect infestation in both accessions of *A. halleri* plants

**Fig. 6:**
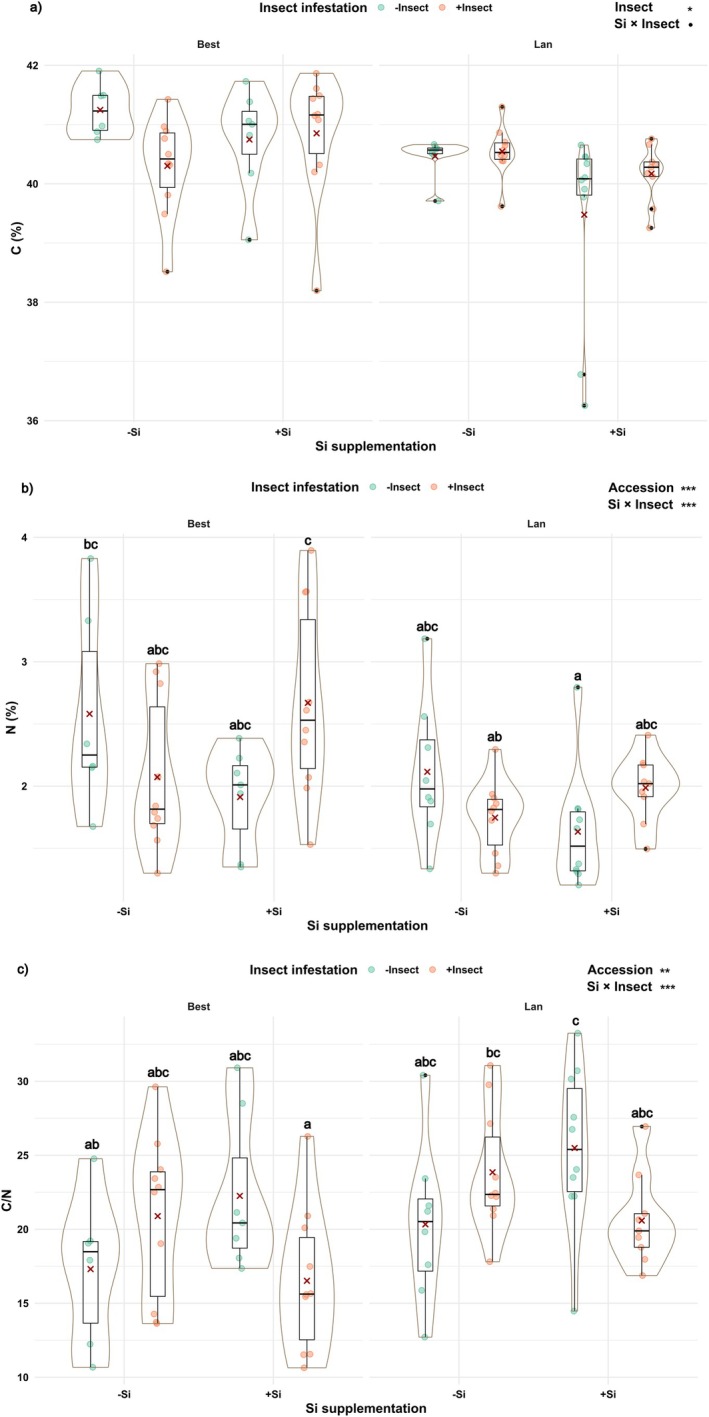
Shoot contents of (a) carbon (C), (b) nitrogen (N) and (c) C to N ratio of *Arabidopsis halleri* plant accessions [Bestwig (Best) and Langelsheim (Lan)] grown on soil without (−Si) or with Si (+Si) supplementation and kept uninfested (−Insect) or infested (+Insect) by *Phaedon cochleariae* larvae. Solid circles represent data points; solid dots, outliers; solid lines, medians; crosses, the means; boxes, the interquartile ranges; and whiskers, the 5% and 95% percentiles surrounded by violin plots indicating the width of data distribution. Statistically significant outcomes are shown as follows: ***P < 0.001, **P < 0.01, *P < 0.05, •*P* < 0.1 (marginally significant). Different letters above the plots indicate significant differences based on Tukey's HSD post hoc test.

Insect infestation led to reduced C contents in plants of the Best accession, but enhanced C contents in plants of the Lan accession (Fig. 6a; Table 1). Accession had a significant impact on shoot N (Fig. 6b; Table 1), whereby N contents were on average 1.2 times higher in plants of the Best than of the Lan. Moreover, they were also affected by Si supplementation × insect infestation with similar patterns in both accessions (Fig. 6b). Insect infestation led to lower N contents in −Si plants but higher N contents in +Si plants, reflecting the ability of +Si plants to maintain shoot N following herbivory. Accordingly, shoot C/N was significantly influenced by accession (Fig. 6c; Table 1), whereby it was on average 1.17 times lower in plants of the Best than in plants of the Lan. Si supplementation × insect infestation also impacted C/N significantly in plants of both accessions, with an increased ratio in −Si plants, but a decreased ratio in +Si plants, in response to herbivory (Fig. 6c).

### The composition and concentration of shoot glucosinolates were largely affected by accession × insect infestation

**Fig. 7:**
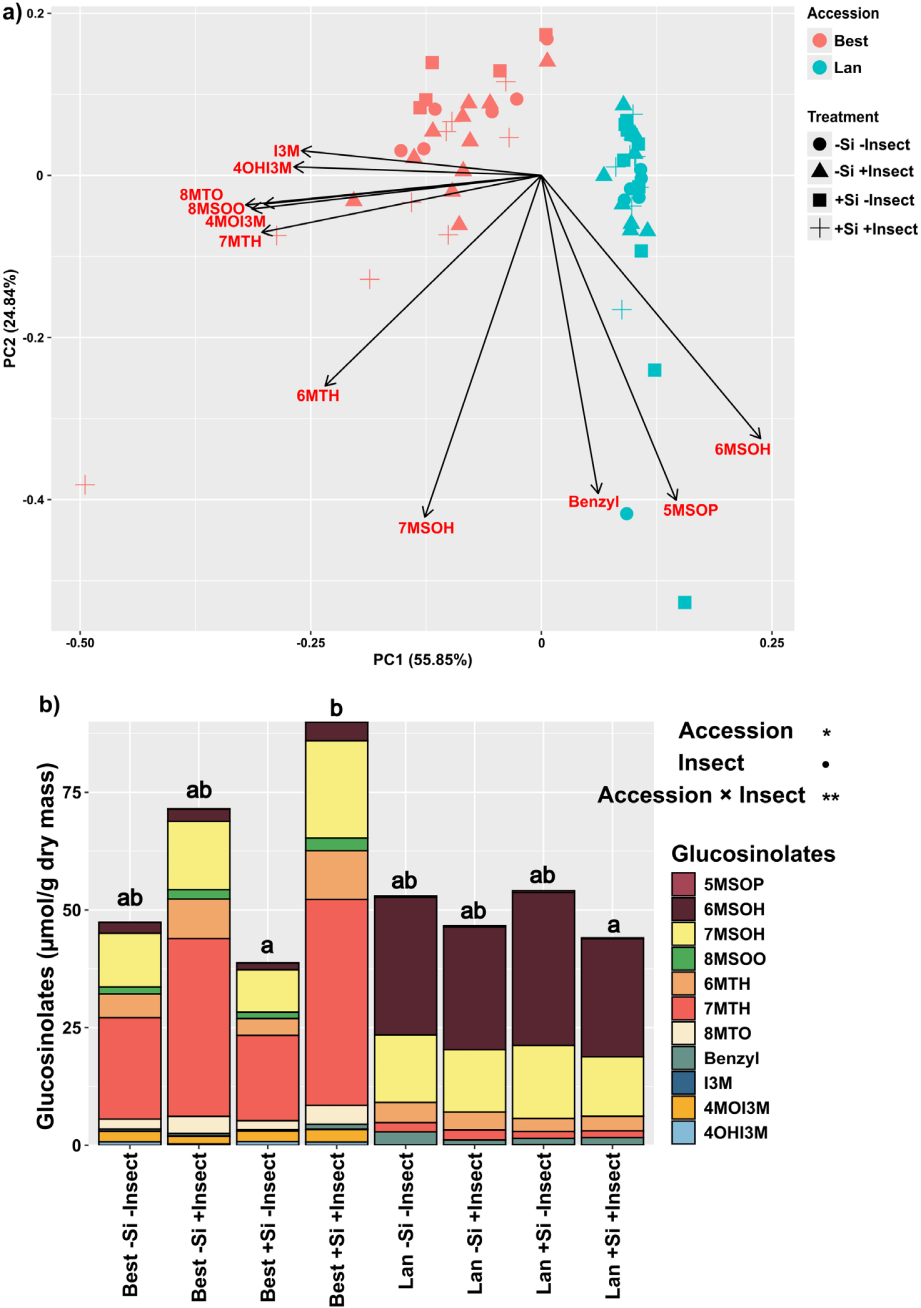
(a) Score plot of principal component analysis (PCA) with scores and loadings are indicated by coloured symbols and arrows, respectively, and (b) composition of 11 glucosinolates based on mean concentrations (μmol/g dry mass) found in shoots of two accessions (Best and Lan) of *Arabidopsis halleri* plants grown on soil without (−Si) or with Si (+Si) supplementation and kept uninfested (−Insect) or infested (+Insect) by *Phaedon cochleariae* larvae. Glucosinolates are abbreviated as follows: 5MSOP (5‐methylsulfinylpentyl glucosinolate); 6MSOH (6‐methylsulfinylhexyl glucosinolate); 7MSOH (7‐methylsulfinylheptyl glucosinolate); 8MSOO (8‐methylsulfinyloctyl glucosinolate); 6MTH (6‐methylthiohexyl glucosinolate); 7MTH (7‐methylthioheptyl glucosinolate); 8MTO (8‐methylthiooctyl glucosinolate); I3M (indol‐3‐ylmethyl glucosinolate); 4MOI3M (4‐methoxyindol‐3‐ylmethyl glucosinolate); and 4OHI3M (4‐hydroxyindol‐3‐ylmethyl glucosinolate). Statistically significant outcomes of the total concentrations of shoot glucosinolates are shown as follows: **P < 0.01, *P < 0.05, •*P* < 0.1 (marginally significant).

In total, 11 glucosinolates could be detected in shoots of *A. halleri* plants, of which seven were aliphatic, one benzenic and three indolic glucosinolates. While all glucosinolates were present in plants of the Best accession, 8‐methylthiooctyl glucosinolate (8MTO) and indol‐3‐ylmethyl glucosinolate (I3M) were not detected in shoots of the Lan accession. The first two components of the PCA explained 80.7% of the variance of the composition of shoot glucosinolates and showed a distinct separation between accession (df = 1, *F* = 140.42, *P* = 0.001), insect infestation (df = 1, *F* = 4.07, *P* = 0.03) and their interaction (df = 1, *F* = 7.04, *P* = 0.002) based on a PERMANOVA (Fig. 7a).

Shoots of the Best accession contained substantial concentrations of 7‐methylthioheptyl glucosinolate (7MTH) (Fig. S1, Table 1), but low concentrations of 6‐methylsulfinylhexyl glucosinolate (6MSOH), while these patterns were the other way around in shoots of the Lan accession (Fig. 7b). Moreover, shoots of the Best accession also contained substantial concentrations of 8‐methylsulfinyloctyl glucosinolate (8MSOO) and 8MTO. In contrast, shoots of the Lan accession contained threefold higher concentrations of benzyl glucosinolate than shoots of the Best accession (Fig. 7b). The total concentration of all glucosinolates was on average 1.25 times higher in shoots of the Best than in those of the Lan accession (Fig. 7b; Table 1).

The shoot concentrations of most glucosinolates, except for 4‐methoxyindol‐3‐ylmethyl glucosinolate (4MOI3M), were influenced by the experimental factors in both accessions (Table 1). In particular, shoot concentrations of 7‐methylsulfinylheptyl glucosinolate (7MSOH), 8MSOO, 6‐methylthiohexyl glucosinolate (6MTH), 7MTH and 8MTO were significantly influenced by accession × insect infestation, being more induced by herbivory in the Best than in the Lan (Fig. 7b; Fig. S1; Table 1). Furthermore, there were significant three‐way interactions between concentrations of 5‐methylsulfinylpentyl glucosinolate (5MSOP) and 6MSOH (Table 1). Benzyl glucosinolate had similar mean concentrations between −Insect and +Insect in −Si plants, but significantly lower concentrations in −Insect than +Insect +Si plants (Fig. 7b; Table 1). Shoot concentrations of two indolic glucosinolates, that is I3M and hydroxyindol‐3‐ylmethyl glucosinolate (4OHI3M), both increased by 88.5% and decreased by 31.3% in response to Si supplementation and insect infestation, respectively, but independently (Fig. 7b; Table 1).

## DISCUSSION

We show that shoot Si concentrations were not induced by insect infestation in both accessions of *A. halleri*, in contrast to previous studies reporting Si inducibility by insect herbivory in some Poales species (Johnson *et al*. 2020; Cibils‐Stewart *et al*. 2023). In low Si‐accumulating species, such as *A. halleri*, Si may not be a potent elemental defence, and thus, Si inducibility may not be expected when plants are attacked by an insect herbivore. Recent studies reported that Si‐based defences were also not induced following herbivory by caterpillars of the generalist *Spodoptera frugiperda* (J. E. Smith, 1797) (Lepidoptera: Noctuidae) in some tropical tree species (Araliaceae, Clusiaceae, Fabaceae, Malvaceae, Moraceae, Myrtaceae and Rubiaceae) (Klotz *et al*. 2023) and *Spodoptera littoralis* (Boisduval, 1833) (Lepidoptera: Noctuidae) in a Fabaceae species (Putra *et al*. 2024).

Plant resistance against *P. cochleariae* larvae was not influenced by Si supplementation. This may be explained by the fact that our +Si *A. halleri* plants accumulated relatively low concentrations of shoot Si (less than 1% dry mass), which may not be sufficient to provide resistance. The biological significance of Si in low Si‐accumulating plant species is still poorly understood in comparison with that in high Si‐accumulating plant species (Putra *et al*. 2020). A previous study reported that low concentrations of Si deposits in the form of phytolith (silica body) in some plant species of southwest Asian Asteraceae poorly acted as anti‐herbivore defences against medium‐to‐large mammalian herbivores (Katz *et al*. 2014). However, in other low Si‐accumulating species, such as *Arabidopsis thaliana*, Si supplementation conferred protection against a powdery mildew fungal pathogen *Erysiphe cichoracearum* relative to plants without Si supplementation (Fauteux *et al*. 2006). Protection against viruses was also shown in tobacco, *Nicotiana tabacum* (Solanaceae), when hydroponically grown in +Si compared with those in −Si solution (Zellner *et al*. 2011). Besides these findings, Si may enhance symbiotic functions in some low Si‐accumulating Fabaceae species associated with N‐fixing rhizobia (Johnson *et al*. 2017; Putra *et al*. 2021, 2022). In other words, the low plant Si concentration *per se* may not necessarily imply an insignificant role of Si in the alleviation of some environmental stresses (Katz 2014). For protection against herbivorous insects in particular, other metals, such as Cd and Zn, could be more relevant for *A. halleri*; a previous study demonstrated that Cd and Zn hyperaccumulation in leaves of this plant effectively deterred specialist herbivorous insects, including *P. cochleariae* adults (Kazemi‐Dinan *et al*. 2014).

Interestingly, we found that plants of the Lan accession were more resistant than those of the Best accession against *P. cochleariae* despite the latter having higher concentrations of shoot glucosinolates. This insect species is able to cope well with the glucosinolate–myrosinase system of Brassicaceae by conjugating toxic glucosinolate‐derived metabolites with aspartic acid (Friedrichs *et al*. 2020; Barber *et al*. 2024). Instead of glucosinolates, differences in other metabolite classes or in mechanical defences between plants of these two accessions may be more relevant for resistance against this specialist insect. This is indicated by the fact that in the paired‐choice bioassays, *P. cochleariae* larvae preferred feeding on leaf discs from the Best over the Lan accession. Leaves of the latter were found to have a significantly higher (non‐glandular) trichome density than leaves of the Best accession. In *A. halleri* subsp. *gemmifera*, a trichome polymorphism, with (hairy) or without (glabrous) trichomes, was found in two populations (Honjo & Kudoh 2019). Apparently, hairy plants experienced less folivory by the leaf beetle species *Phaedon brassicae* native to East Asia, particularly when glabrous plants were abundant, suggesting a potential frequency‐dependence in the field (Sato *et al*. 2014). However, leaf glucosinolate concentrations of these *A. halleri* plants did not significantly differ (Sato *et al*. 2014). In our present study, potential ecological or evolutionary drivers that may act as selection pressures on mechanical defences in leaves of the Best and Lan accessions deserve further investigations, especially at their local habitats. Best and Lan plants are about 335 km geographically apart within Germany, where they face specific environmental conditions. For example, plants of the Best accession grow mostly under the tree canopy, while in contrast, plants of the Lan accession grow predominantly in an open habitat (R. Putra, personal observation). Potentially, plant canopy in those locations may influence UV‐B penetration and exposure to the plants, which may have consequences for the leaf trichome density (Honjo & Kudoh 2019; Karabourniotis *et al*. 2020).

Effects of Si supplementation on shoot C contents are well documented in Poaceae, whereby a higher accumulation of Si often leads to decreases in C contents, corroborating the trade‐off hypothesis between Si and C (Johnson & Hartley 2018; Hodson & Guppy 2022). Our results, performed with *A. halleri*, are in line with this hypothesis, particularly in +Si plants of the Lan accession. However, in plants of the Best accession, C contents were slightly lower in insect‐infested than in non‐infested −Si plants, but were quite similar between the +Si plants of both insect treatments. Decreased C contents, as found in the −Si plants, may be related to a disruption of the photosynthetic apparatus due to tissue losses by insect herbivory (Nykänen & Koricheva 2004). Moreover, we showed that there was a significant effect of Si × herbivory on contents of shoot N in both accessions. In −Si individuals, N contents were significantly lower in +Insect than in −Insect plants, but this pattern was the other way round in +Si individuals. These results indicate that +Si plants may become more flexible than −Si plants to allocate C and N in protection against a biotic stressor, potentially via the production of defensive metabolites, including phenolics and glucosinolates (Omirou *et al*. 2009; Schultz *et al*. 2013). Our consistent outcomes of insect performance and feeding preference as a proxy for plant resistance in both experiments may also be related to a distinct resource allocation of shoot C/N in both plant accessions. Larvae of *P. cochleariae* may be negatively impacted by a higher shoot C/N in plants of the Lan, which might indicate increased other C‐based organic defences besides glucosinolates, for example some phenolics, including flavonoids in tomato (Royer *et al*. 2013).

The glucosinolate profiles in plants of the Best and Lan accessions displayed two distinct chemotypes, as previously described for these two accessions (Kazemi‐Dinan *et al*. 2015). Besides being constitutively produced, glucosinolates are also known to be inducible by insect infestation (Textor & Gershenzon 2009). As either constitutive or induced defences, maintenance of distinct glucosinolate chemotypes may be driven by different biotic pressures from insect herbivory, as, for example, found among wild cabbage, *Brassica oleracea* (Newton *et al*. 2010). In previous work on *A. halleri* plants, differences in glucosinolate profiles could be related to metal concentrations in the soil, such as Cd and Zn, and other edaphic factors, such as pH, N and S availability (Kazemi‐Dinan *et al*. 2015; Putra *et al*. 2024). Soil physicochemical aspects alone could explain the plasticity of glucosinolate profiles in another Brassicaceae, *Boechera stricta* (Wagner & Mitchell‐Olds 2023). In our study, concentrations of five glucosinolates, that is 7MSOH, 8MSOO, 6MTH, 7MTH and 8MTO, as well as total glucosinolate concentrations were significantly enhanced by insect herbivory in plants of the Best accession. Interestingly, among them, 8MSOO, 7MTH and 8MTO are characteristics of the chemotype of this accession (Kazemi‐Dinan *et al*. 2015; Putra *et al*. 2024). These glucosinolates may protect plants from generalist herbivores (Textor & Gershenzon 2009). In contrast, several specialists, including *P. cochleariae*, have been shown to be stimulated for feeding by glucosinolates (Tanton 1977; Reifenrath & Müller 2008). Concentrations of two indolic glucosinolates, I3M and 4OHI3M, decreased following herbivory by *P. cochleariae* larvae in plants of the Best accession. Likewise, reduced concentrations of I3M were found in leaves of the Lan accession in response to an infestation by a generalist aphid, *Myzus persicae* (Stolpe *et al*. 2017).

We also found an interactive effect of Si supplementation and other factors, such as plant accession and/or insect infestation, on 5MSOP, 6MSOH and benzyl glucosinolates. Once accumulated in plant tissues, Si has been shown to be immobile, largely uncharged and unreactive at physiological pH or chemically inert (Coskun *et al*. 2019). These traits may hinder Si from directly binding with many other atoms, as opposed to C (Coskun *et al*. 2019), to form organic metabolites. Considering this, it is unlikely that Si directly interferes with glucosinolate metabolism in *A. halleri*. However, Si may indirectly affect this specialised metabolism in two ways: First, we found that Si supplementation significantly impacted the C and N resource allocation in shoots. A change in shoot C and N contents could then potentially shift the metabolism of certain glucosinolates. Second, Si has been presumed to act as a priming stimulus in *B. distachyon*, where +Si plants showed a quicker JA induction following herbivory (Hall *et al*. 2019). If that is the case, a potential induction of JA in +Si plants could also lead to increases in some specialised defensive metabolites, including glucosinolates (Doughty *et al*. 1995). Further studies are needed to test this hypothesis.

## CONCLUSION

Our work demonstrates that plant resistance of *A. halleri* against a specialist beetle species is more likely influenced by the genotypic variation in defence strategies of the two accessions, that is Best and Lan. This variation is reflected in the higher concentration of glucosinolates (organic defence), but much lower foliar trichome density (mechanical defence) in the former. However, both plant accessions significantly accumulate Si when supplemented with Si (+Si plants). This means that while plants responded to Si effectively, increased Si in the plants might not be functionally regarded as an elemental defence *per se* in *A. halleri*. Instead, Si supplementation may contribute to support the development of foliar trichomes in plants of the Lan accession. Distinct genetic backgrounds of the plant species and available Si in the soil may be important components, which could further impact the performance and preference of *P. cochleariae* larvae feeding on these plants.

## AUTHOR CONTRIBUTIONS

RP and CM conceptualised the idea, designed the experiments and revised the manuscript. RP conducted Experiment 1 together with MP, conducted Experiment 2 and analysed all data from both experiments, and led the writing of the manuscript. CM gave detailed comments on the manuscript. All authors agreed to submit the final version of the manuscript.

## Supporting information


**Fig. S1.** Shoot concentrations of 7MTH (7‐methylthioheptyl glucosinolate) of *Arabidopsis halleri* plant accessions (Best and Lan) grown on soil without (−Si) or with Si (+Si) supplementation and kept uninfested (−Insect) or infested (+Insect) by *Phaedon cochleariae* larvae. Solid circles represent data points, solid dots outliers, solid lines medians, crosses the means, boxes the interquartile ranges and whiskers the 5% and 95% percentiles surrounded by violin plots indicating the width of data distribution. Statistically significant outcomes are shown as follows: ***P < 0.001, **P < 0.01, *P < 0.05. Different letters above the plots indicate significant differences based on the Tukey's HSD post hoc test.
