# Supplementary figures and images for: Plant accession and insect infestation, rather than silicon supplementation, shape defence strategies of Arabidopsis halleri towards a leaf beetle
Source: Plant Biol (Stuttg). 2025 Dec 22;28(2):407–19. doi: 10.1111/plb.70160 (PMC12884025; doi:10.1111/plb.70160)

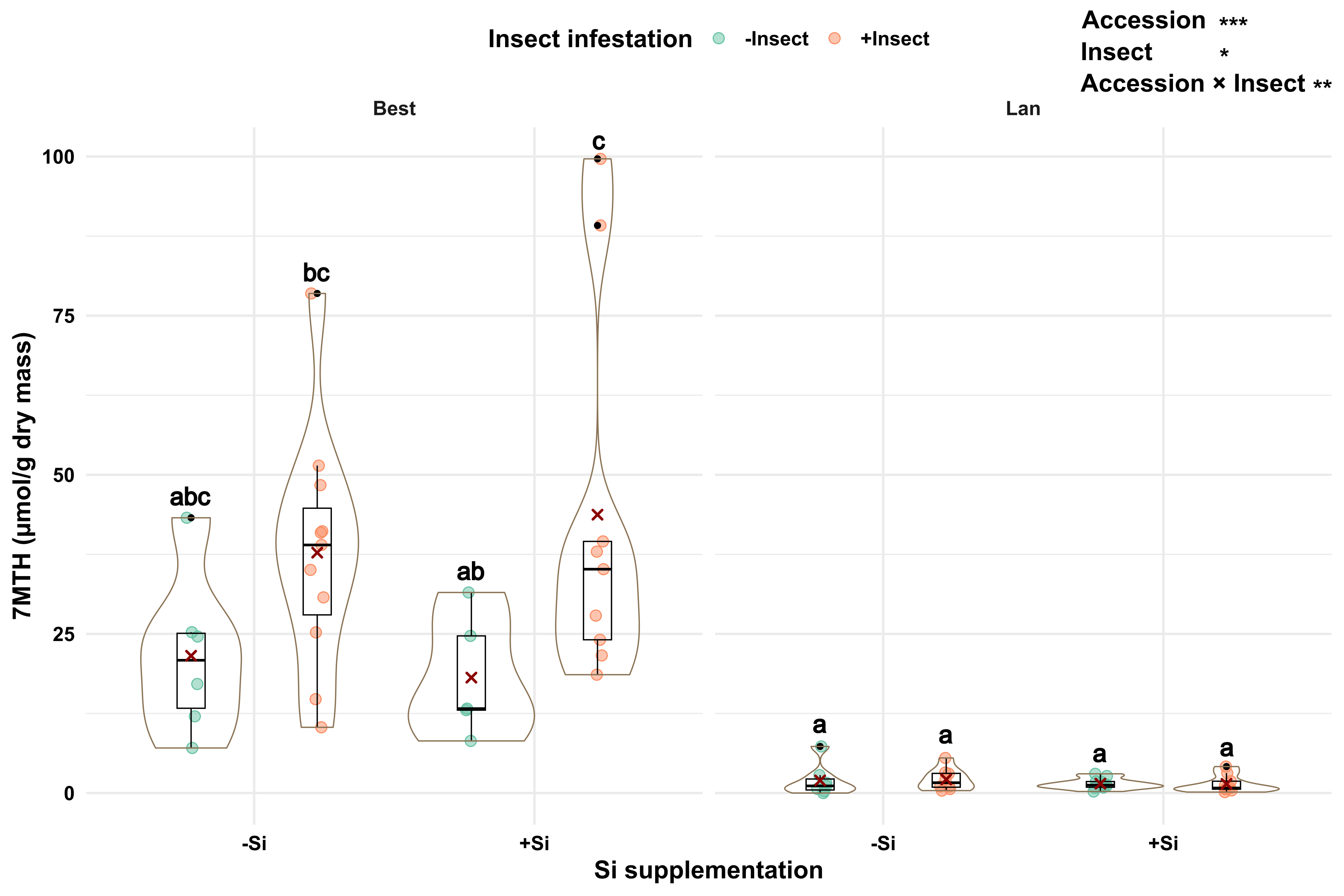

Supplement: Supplementary file 1 — Fig. S1. Shoot concentrations of 7MTH (7‐methylthioheptyl glucosinolate) of Arabidopsis halleri plant accessions (Best and Lan) grown on soil without (−Si) or with Si (+Si) supplementation and kept uninfested (−Insect) or infested (+Insect) by Phaedon cochleariae larvae. Solid circles represent data points, solid dots outliers, solid lines medians, crosses the means, boxes the interquartile ranges and whiskers the 5% and 95% percentiles surrounded by violin plots indicating the width of data distribution. Statistically significant outcomes are shown as follows: ***P < 0.001, **P < 0.01, *P < 0.05. Different letters above the plots indicate significant differences based on the Tukey's HSD post hoc test. [file PLB-28-407-s001.png]
